# Flourishing, religion, and burnout among caregivers working in pediatric palliative care

**DOI:** 10.1177/00912174241229926

**Published:** 2024-01-27

**Authors:** Annemarie E. Oberholzer, Benjamin R. Doolittle

**Affiliations:** 1 Department of Christian Spirituality, 56866 University of South Africa (UNISA), Pretoria, South Africa; 2 Internal Medicine & Pediatrics, 12228 Yale School of Medicine, New Haven, CT, USA

**Keywords:** flourishing, religion, burnout, caregivers, pediatric palliative care, meaning and purpose

## Abstract

**Introduction:**

Providers working with children who are dying are especially prone to burnout. The enhancement of human flourishing in providers may mitigate burnout and improve quality of care. However, the relationship between job satisfaction and human flourishing has not been well studied. This project explores factors that promote human flourishing among caregivers working with children in pediatric palliative care in South Africa.

**Methods:**

A convenience sample of caregivers working in pediatric palliative care were invited to complete an anonymous, confidential survey . The survey also included open-ended questions to explore opinions and attitudes about job satisfaction, struggles, and coping.

**Results:**

Twenty-nine people from a variety of occupations and work environments completed the survey. The prevalence of burnout was 3/29 (10%). Life satisfaction (an indicator of flourishing) was associated with private religious activities (*r* = .38, *P* < .05), and carrying religion into all aspects of life (*r* = .44, *P* < .05). Burnout was not associated with life satisfaction, although power was limited. Qualitative analysis of open-ended questions revealed the following themes as factors contributing to satisfaction at work (flourishing): being able to make a difference, finding meaning and purpose, having a relationship with the children and their families, and working within the context of a multi-disciplinary team. A number of challenges to this work were also identified, including lack of resources, problems within the team, and the emotional demands of care.

**Conclusions:**

Despite job stress and the difficult work of caring for terminally ill children, several factors were associated with flourishing. These findings may help to enhance the flourishing of caregivers in the resource-challenged setting of pediatric palliative care in South Africa.

## Introduction

There are few caregivers who are willing to walk with children through their terminal illness, but what motivates these people to do what they do. Is it possible that they can flourish even as they walk along children who are dying? The care of terminally ill children is particularly challenging among front-line caregivers. Amery et al caution that, “dealing with dying children and their families means sharing a painful and heavy burden, and occasionally that burden can begin to affect us, our work and ultimately our happiness itself.”^1^ Burnout is defined as a constellation of symptoms which include emotional exhaustion, depersonalization, and low personal accomplishment and associated with worse mental health, increased medical errors, and other systemic challenges in healthcare workers.^2^ However, burnout among palliative care providers varies widely. A systematic review by Dijxhoorn et al^3^ reports a prevalence of burnout as low as 3% among a cohort of clinics in the United States^4^ to as high as 66% among French palliative care clinicians.^5^ Prevalence of burnout among palliative care nurses ranges between 24-30%.^6^

Yet despite the extensive literature on burnout among healthcare workers, there is a notable gap in our understanding of models of human flourishing and job satisfaction among palliative care providers, particularly those working with terminally ill children. Gielissen et al explored positive aspects of human flourishing and thriving among primary care physicians, describing it as “an important, untapped resource to better inform physician well-being.”^7^ Seligman describes the following elements as contributing to well-being: positive emotion; engagement; meaning; positive relationships; and accomplishment.^8^ In a comprehensive review, Mishra et al concluded that those with high religiosity have lower rates of depression, improved physical health, and even longer lifespan.^9^

It is therefore important to explore these factors in the context of pediatric palliative care in South Africa. Rensburg describes the healthcare system in South Africa as “two-tiered, and highly unequal,” with a state-funded public sector that suffers from insufficient funding, while the majority of South Africans are unable to afford the high expenses associated with private healthcare.^10^ Only 17% of South Africans has medical insurance and can afford private healthcare, while as many as 45 million must rely on public healthcare.^11^ The public healthcare sector also faces a shortage of all healthcare professionals, with a ratio of .8 doctors per 1000 patients.^12^ Workers in palliative care in South Africa encounter additional challenges, such as lack of resources and historical racial stigma, political challenges, and inter-cultural issues.^13^ Given the complex healthcare system, the lack of resources, and the difficult emotional work, this project’s main objective was to explore aspects of human flourishing and burnout among healthcare workers devoted to terminally ill children by asking the following questions:

What is the level of human flourishing that the caregivers experience?

What role does religion play in their lives?

What is the likelihood of burnout in these caregivers?

To what extent do they find meaning and purpose in their work and in life in general?

## Methods

### Study design

During this study, data were captured from a quantitative questionnaire, followed by qualitative open-ended questions. Koenig explains that this approach is helpful to qualitatively interpret the data that is collected through a quantitative method.^14^ In this study, it is used to explain in more depth the flourishing - and specifically elements of meaning and purpose - of caregivers working with terminally ill children in South Africa. Koenig also suggests that, when participants are encouraged to write about their experiences, it could lead to more optimal data as participants get the opportunity to reflect on their own answers.^14^

### Data collection

Due to the small number of caregivers working in pediatric palliative care in South Africa, purposive sampling was used to identify caregivers such as healthcare providers and counsellors working with children at the end of life throughout South Africa. Information was collected by means of an online questionnaire, consisting of 3 parts: the demographic data of the participants, a quantitative questionnaire, as well as a qualitative questionnaire.

The quantitative questionnaire consists of 3 instruments. The *Flourishing Measure* is a validated instrument that explores the following 6 domains: life satisfaction; mental and physical health; meaning and purpose; character and virtue; social relationships; and financial and material stability. This instrument is used widely to evaluate holistic well-being in cross-cultural contexts, and each item is scored on a 0-10 scale.^15^ A burnout assessment was done with the single item measures of emotional exhaustion (“I feel burned out from my work”) and depersonalization (“I have become more callous towards people since I took this job”), which have strong correlations with the 22 item Maslach Burnout Inventory.^16^ The two-question version of the Maslach is used widely in large population studies to evaluate the burnout syndrome.^16^ The *Duke University Religion Index* (DUREL) is another validated instrument that was used to establish the personal importance of religion for the participant, consisting of 5 items which explore Organizational Religious Activities (ORA); Non-organizational Religious Activities (NORA); and Intrinsic Religiosity (see Table 1). Similarly, the DUREL has been widely used in hundreds of studies to evaluate the multi-dimentional nature of religious observance.^17^ Because of the small sample size, the quantitative questionnaires provide insufficient data to be interpreted on its own. It allows, however, for a deeper understanding of the research questions and sheds more light on the qualitative aspect of the study.

**Table 1.** Correlation of DUREL and flourishing instruments.

DUREL domain	Questions	Flourishing (average) Spearman’s rank correlation	*P* value
Organizational religious activities (ORA)	How often do you attend church or other religious meetings?	.28	NS
Non-organizational religious activities (NORA)	How often do you spend time in private religious activities, such as prayer, meditation or bible study?	.38	<.05
Intrinsic religiosity	In my life, I experience the presence of the divine.	.34	NS
	My religious beliefs are what really lie behind my whole approach to life.	.25	NS
	I try hard to carry my religion over into all other dealings in life.	.44	<.05
	Intrinsic-total.	.42	<.05

The qualitative questionnaire consists of the following open-ended questions: Do you find joy in your work? If so, what gives you joy? What is the biggest challenge in your work? And why did you choose to work with terminally ill children? Participants were also given another chance to elaborate or comment on any of the previous questions asked.

### Data analysis

Given the small sample size, Spearman rank correlation coefficients were calculated for continuous variables to correlate the DUREL and Flourishing Index. Student t-test was performed to evaluate the dichotomous variable of the burnout syndrome with the DUREL and the Flourishing Measure. Statistical analysis was performed using JMP software.^18^

Qualitative data was analyzed according to the framework method as explained by Gale et al^19^ During analysis of the interview data, the data were described using the subjective ideas and expressions used by the participants. Themes were formed and expanded upon by examining data categories and comparing different data sets. This was then discussed among the researchers in order to reach an agreement.

### Ethical considerations

This project adhered to ethical principles of informed consent, limiting any risk of harm, and maintaining confidentiality and anonymity. A link to access the questionnaires was emailed to prospective participants where they had the option to complete the questionnaires anonymously. The purpose and objectives of the study were explained and informed consent obtained at the beginning of the questionnaires.

This study received ethical clearance from the College of Human Sciences Research Ethics Review Committee at UNISA (CREC Reference number: 90315758_CREC_CHS_2022).

## Results

### Quantitative analyses

Twenty-nine participants from across South Africa completed the survey. Table 2 describes the demographic characteristics of the participants.

**Table 2.** Demographic data of participants (n = 29).

Occupation	Medical doctor (MD)	11
	Nurse (RN)	4
	Social worker (SW)	4
	Spiritual counsellor (SC)	1
	Speech therapist (ST)	1
	Physiotherapist (PT)	1
	Program coordinator (PC)	4
	Manager (M)	3
Workplace	Public hospital (PubH)	16
	Private hospital (PrivH)	3
	Non-profit organisation (NPO)	6
	Hospice	3
	No response	1
Patient population	Terminally ill and dying children only	3
	Terminally ill and dying children as well as children who recover/go into remission and are discharged	26
Remuneration	Paid position	27
	Volunteer	2
Religion	Christian	15
	Islam	3
	Hindu	1
	Christian and buddhist	1
	Spiritual but not religious	2
	No religion	5
	Didn’t answer	2
Work hours per month	Minimum	5 h
	Maximum	360 h
	Average	150 h
Years of experience	Minimum	1 years
	Maximum	27 years
	Total	360 years

**Figure 1.**
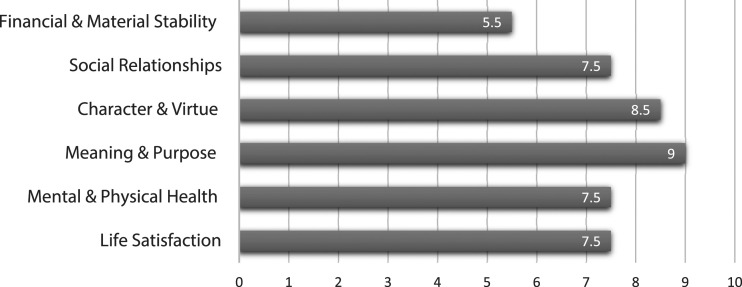
Flourishing data.

In the Flourishing Measure, the participants scored an average of 5.5 (sd +/− 2.8) for financial and material security, 7.5 (sd +/−1.7) for social relationships, 8.5 (sd +/− 1.3) for character and virture, 9.0 (sd +/− 1.8) for meaning and purpose, 7.5 (sd +/− 1.5) for mental and physical health, and 7.5 (+/− 1.4) for life satisfaction (see Figure 1).

Based on the two questions MBI, 3/29 (10%) participants met criteria for burnout. Those who were experiencing burnout did not have significantly different scores on the DUREL or the Flourishing Index. There were several items in the DUREL which had statistical correlation with life satisfaction, an item in the Flourishing Index (Table 1). Life satisfaction was correlated with NORA (*P* = .38, *P* < .05), “carrying my religion into all other dealings in life,” (*P* = .44, *P* < .05), and the total intrinsic religiosity score (*P* = .42, *P* < .05).

### Qualitative analyses

Participants participated enthusiastically and gave detailed reports on the open-ended questions which will now be discussed. Please refer to Table 2 for clarification of abbreviations used. A summary of the themes that emerged can be found in Table 3.

**Table 3.** Themes from qualitative questions.

Question	Themes	Sub-themes
Do you find joy in your work? If so, what gives you joy?	Making a difference	Experiencing a positive outcome
		Advocate for change
	Having meaning and purpose	Work is a calling
		Fulfilment
	Relationship with children and families	Being inspired by the children
		Connecting with the child and family
	Interaction with the team	Able to teach and influence others
		Being inspired by others
What is the biggest challenge in your work?	Work team	Lack of knowledge
		Bureaucracy
	Emotional demanding	Emotions surrounding the death of a child/baby
		Conflicting beliefs
	Lack of resources	Funding
		Human resources
	Time constraints	Time for patients
		Time for self-care and development
Why did you choose to work with terminally ill children?	Calling	Divine purpose
	Personal experience	Family member
		Self
	Rewarding	Making a difference

### Do you find joy in your work?

Most participants find joy in their work, but some remarked that “satisfaction” is a better word than “joy.” The following themes emerged.

#### Being able to make a difference

Participants referred to the joy of a positive outcome and being able to make a difference. One respondent said that “hearing a child’s laughter lifts your spirit and gives you energy to carry on” (MD, PubH), and another mentioned the joy of “helping someone who lost hope” (SC, PubH). Participants also mentioned talking to children about their impending death: “…for those who do not recover, just chatting about heaven and the joys of playing in the fields there or the possibility of seeing passed friends or family” (PC, PubH). A second sub-theme that emerged was being able to advocate for change: “Having advocated for a terminally ill child or ensuring we allow a child to die in dignity, pain free and taking away all suffering … gives me joy” (MD, PubH).

#### Finding meaning and purpose

Participants described finding meaning and purpose in their work. Several participants referred to their work as a “calling,” and “knowing I am exactly where I am supposed to be” (MD, PubH). They also find fulfilment in their work, and 1 commented on “the satisfaction of serving” (MD, PubH).

#### The relationship with the children and their families

Several participants mentioned that the children inspire them. Participants stated that “these children have taught me more about life, love, hope and never giving up than I could have dreamed of” (M, NPO), and that “the joy comes specifically from the children’s resilience” (SW, PubH). Another mentioned that “I would still do this line of work if I wasn’t paid a stipend…. visiting the hospitals and the children, almost always encourages me to rise above my worries because they rise above their struggles with little to no complaints” (PC, NPO). Participants also commented on being able to “become part of the family” (MD, PubH). One mentioned the joy of having a “true connection… being able to relate with a shared spiritual experience” (MD, PubH).

#### The interaction with their team

Participants found joy in being part of a multidisciplinary team where they can learn from each other, and it brings joy “when a doctor or nurse finally gets the meaning of palliative care” (MD, PrivH). However, they are also being encouraged by others: “My work team also inspires me tremendously!” (MD, NPO).

### What is the biggest challenge in your work?

The following themes were identified as challenges in the work environment.

#### The work team

Just as participants commented that it could be joyful to be part of a multidisciplinary team, they also commented that their team poses certain difficulties. The first sub-theme that emerged was a lack of knowledge of team members: “When colleagues …want to persist with all treatment despite me asking what we are trying to achieve, what quality of life are we giving to this child… Are we treating our conscience or are we treating the child?” (MD, PubH), and a “complete lack of understanding of holistic care” (MD, PubH). Bureaucracy in the system is also difficult to deal with: “… the thing that would make me not sleep at night is the poor administration” (MD, PubH). One respondent also commented on the “poor communication within teams … our voices are often not heard” (ST, PubH).

#### The emotional demanding aspect of the work

The emotions surrounding the death of a child is very difficult to deal with, and 1 participant referred to “the sadness and the emotional trauma when a child dies” (M, NPO). Another confessed that “I’m still struggling to face death as it really breaks me down” (SW, hospice), and the “grief and raw emotions of the family when a child dies” is also challenging (RN, PrivH). The second sub-theme that emerged was that the conflicting beliefs of the family are often a challenge to deal with: “having to make family understand that it is medical, that is why the child is ill, and not related to tradition or ancestorial beliefs” (MD, PubH).

#### A lack of resources

Several participants commented on the lack of resources, stating that “We are always on the edge when it comes to job security and funding” (MD, NPO), and “the low salaries paid to staff” (RN, hospice). Not only is funding a problem, but human resources are also a challenge: “The clinical workload is immense for our complement of staff. An equivalent unit in the United States, serving the same population number, would have up to 10 times more staff than we have!” (MD, PubH).

#### Time constraints

Several participants mentioned that time constraints are a big challenge. The first sub-theme that emerged is that there is not enough time to spend with the child and family, and a lack of human resources and late diagnoses were mentioned as reasons for this: “There are just too few of us to spend the time with these children and their families that they need and deserve” (RN, PubH); and, “late notification means late diagnosis so there is no chance to build up a friendship” (PC, PubH). On the other hand, participants also reported that here is not enough time for self-care and development: “Self-care extremely important, it is not a luxury, it is a necessity” (MD, PubH); and, “finding time to devote to expanding services and conducting research” (MD, PubH).

### Why did you choose to work with terminally ill children?

When asked what the reason is for them to be working with terminally ill children, the following themes emerged: a calling; a previous experience; and a rewarding vocation.

#### A calling

Several participants remarked that their work is a divine calling: “I believe this is where God wants me to be, fulfilling His purpose for my life” (RN, PrivH); and, “I am part of a greater plan where I have a role to play in whatever way is fit” (M, NPO).

#### Personal experience

Some participants ended up in this field because they have personal experience of the field and can relate. “I never knew this concept of palliative care existed having experienced living with my father who had a terminal illness and passed on” (MD, PubH); “my daughter is a childhood cancer survivor” (M, NPO); and, “my son died from cancer” (M, NPO). Others were diagnosed themselves: “I saw children going for radiation when I was undergoing my treatment, so I started visiting the ward, and it all started from there” (PC, PubH).

#### Rewarding

Several participants commented on how rewarding it is to work with terminally ill children, and 1 participant added: “it makes me feel like I make a true difference in a sometimes lonely and confusing time for families” (MD, NPO).

A medical doctor working in a public hospital commented:

Early on in my life as a consultant - I used to say - that every time a child died, a little piece of me died too. Many years later, I understood the privilege I had, in easing the pain, in breaking the bad news in a better way. It still sits very heavily with me, but I see it as a different experience now.

## Discussion

This study explored aspects of flourishing, religiosity, and burnout in caregivers working with terminally ill children, and the results indicate that, despite the challenges they must face, several factors were associated with flourishing. Participants reported high levels of meaning and purpose in the Flourishing Measure that also correlates with the feedback given in the open-ended questions. This is confirmed by other research studies, stating that staff members in a children’s hospice feel that they can make an actual difference^20^ and that oncology nurses found meaning in their work and that it gives them a purpose in life.^21^ McConnell et al cited several studies reporting on healthcare staff “finding meaning and making sense of a child’s death”.^22^ Costin and Vignoles explained that “in evaluating their life’s meaningfulness, most people seemingly think about whether their lives matter beyond the narrowness of their day-to-day existence.”^23^

Participants in the current study reported that the relationship they experience with the children and their families contribute greatly to job satisfaction and experiencing joy in their work. Other authors also confirmed the importance of connecting with patients as a preventative strategy for compassion fatigue^21^ and increased job satisfaction.^7,22^ Participants expressed joy in being part of a multidisciplinary team, and Kase and Doolittle confirms this, stating that social connections in the workplace were identified as an important source of support and comfort during difficult times.^24^

Of note, only 10% of participants met criteria for burnout, far fewer than the average of well-resourced Western countries.^25^ 1 research study concluded that aspects such as meaningful relationships, significant interaction with their patients, as well as their religious beliefs were all identified as contributing factors to thriving in medical residents.^26^ These are all aspects that were found in the current study, and it could possibly be seen as potential protective factors against burnout.

The positive correlation between life satisfaction and religious activities in the current study is an intriguing finding and supported by research. In a review of 18 journals, spiritual and religious beliefs were found to be a way of combatting burnout and an important way for nurses to cope with stress,^27^ and it was also found to be a key aspect to provide a sense of thriving for medical residents.^26^

The struggles with workload as experienced by the participants is not unique. During a review of studies from across the world, McConnell et al referred to several studies confirming that healthcare staff providing end-of-life care to children, experience challenges with regards to workload and time constraints.^22^ In this study, the work team was also identified as a source of stress and a challenge.^20^ Participants sited the lack of knowledge of team members as 1 of their biggest challenges. This is confirmed by Sibomana et al who pointed out that 1 of the barriers that prevents adequate palliative care to children in sub-Saharan Africa is a lack of adequate skills, knowledge, and institutional standards.^28^

However, the well-being and thriving of healthcare providers are important to ensure optimal patient care,^7^ especially to vulnerable populations such as children who are diagnosed with life-limiting and life-threatening conditions. We believe it is therefore important to encourage aspects of flourishing and to include spiritual support for caregivers working in pediatric palliative care.

### Limitations and future directions

There are several limitations to consider. South Africa is a unique country with wide variability of economic resources and these findings may not be generalizable to settings outside of South Africa. However, given the stressors of this unique cohort, these findings offer a compelling model of caregiver thriving. Second, the participants were diverse in their job duties, including physicians, nurses, allied healthcare professionals and non-profit managers. Motivations and perspectives may vary based on the important, albeit different, roles that providers played. This project is therefore not specific to a unique job role as many prior studies performed (ie, nurses or physicians) and captures the unique perspectives of different voices that are not often heard.

Further research to explore these qualities in other healthcare settings would further elucidate this model. In addition to this, future studies looking into the impact of religious and spiritual practices on caregiver well-being, as well as interventions to address the identified challenges, could also provide valuable insights.

## Conclusions

Indeed, “meaning is about mattering,”^23^ and participants in this study reported that they can make a difference in various ways. The relationships formed with the children and families they work with, as well as with their colleagues, sustain them and help them to rise above the challenges they have must face. Palliative caregivers in this resource-limited setting offer a compelling model for human flourishing: shared vision, collegial team-work, and intrinsic spiritual resources, and although the participants were diverse in their job duties, they have a shared mission: the care of terminally ill children that motivate them to find meaning in the work that they do. This united vocation affirms the findings among this diverse cohort, and we would like to end with another quote from 1 of the participants who is a manager at a non-profit organization:

I don’t stress about finance because of the work I do, I would still do this line of work if I wasn’t paid a stipend. Life in general is stressful but I have found that visiting the hospitals and the children almost always encourages me to rise above my worries because they rise above their struggles with little to no complaints.
